# Partial Dentures

**Published:** 1921-10

**Authors:** I. J. Dresch

**Affiliations:** Toledo, Ohio


### PARTIAL DENTURES

BY I. J. DRESCH, TOLEDO, OHIO

Probably, in all prosthetic dentistry there is nothing so generally disliked as partial denture work, and in the same breath we may add, so generally unknown and misunderstood. And yet, when partial work is placed on a scientific basis, the proper technic accurately carried out, partial dentures are no more difficult than full dentures, and quite as satisfactory.

The general attitude toward partial work can be attributed to general misinformation and lack of knowledge of the subject. The average dentist looks upon a partial denture as a make-shift—some-thing that will do until the remaining natural teeth are lost, when a full denture will be made.

If the denture we make will restore lost masticating efficiency, if it will protect the remaining natural teeth from over-functioning, then indeed we will have accomplished something. We will not only restore occlusion, mastication, better assimilation of food, but most important of all, we will save the remaining natural teeth from loss through excessive and unnatural use. A restoration that does these things is worthy of our serious attention, especially when we stop to consider that the average partial denture does not restore occlusion, but in fact usually causes the loss of the abutment teeth.

A partial denture that restores occlusion, automatically relieves the remaining teeth from over-functioning. The problem then is to restore occlusion. To do that we must do more than just set the teeth to come into contact; we must place a denture in the mouth on which the patient will chew.

The requirements for a partial that can be used for masticating purposes are few: first, occlusion; second, tight fit; third, comfort. When these three requirements are combined in a partial denture, the patient will obtain the full benefit of modern dentistry. But if any of these requirements are missing, the effect would be the same as if all of them were missing.

Occlusion, is the first essential, because without occlusion we would not have anything. Suppose then we had occlusion, but the denture was not tight, what would be the effect? Imagine yourself using a partial denture, that when you bite on one side, moves up on the other; when you close on the anterior, it is pushed backward; when you close on the posterior, it is fairly lifted away in front. You might wear such a case, keep it in your mouth, but you would not chew on it. *You would use the remaining natural teeth for mastication,* especially for such effort as is required in cutting fibrous foods, such as meats. When such loose cases are posterior restorations, the anterior teeth soon become in a pathological condition,—then the forceps and a full denture, because nature never intended the anteriors to be used as masticating units. But presuming the denture has occlusion and is tight, but that it is not comfortable, the result is the same. In many cases the denture will be comfortable while the jaws are at rest, but they are not comfortable when the jaws are working.

The chief trouble has been when we made partial dentures tight fitting, we had to sacrifice comfort. The discomfort is usually caused by the tight fitting retaining agent, which places the masticating stress upon the abutment teeth. Then again it is caused by the same agents which tend to grip the abutment teeth in a viselike lock, and cause binding and an uncomfortable tight feeling. In fact when we obtain a rigid connection between the abutment teeth and the restoration, we also have immediate discomfort, because our appliance has the same effect as a class three lever on the abutment tooth.

To thoroughly understand the problem, we must ever keep in mind the fact that the mucosa underlying the denture is displaceable. That when the jaws close on the restoration during mastication, the mucosa is displaced about one millimeter, in the average case. That when this displacement takes place, the denture becomes a third-class lever, prying on the abutment tooth if there is a rigid connection between the two. At the same time we must also bear in mind, that if we do not use a rigid retainer, but use a light band or wire clasp that can move in harmony with the denture, we have trouble from another source.

When the mucosa is displaced the denture becomes a third-class lever, if the denture is rigidly connected to the abutment tooth. A denture one inch long will have a mechanical advantage of nine or ten. If ten pounds pressure is placed at one end the mechanical advantage of the lever will transform this pressure to from ninety to a hundred pounds at the other end. This pressure would not be very destructive if it were placed on the tooth in line with the long axis of the root. But the denture which acts as a third-class lever places this leverage on the tooth at an angle. The pressure or leverage tends to force the tooth from the socket on about the same angle that the forceps act. This movement or stress literally crushes the delicate peridental membrane which is caught between the cementum of the root and the walls of the alveolar process. For as the tooth is pulled to one side, there is no escape for the membrane. That is the reason abutment teeth become sore and sensitive, sometimes actually painful, and without showing any inflammation at the gum margin. When the light clasps move in harmony with the denture, the trouble is caused by friction, for where there is friction there is wear, and when the enamel becomes sufficiently worn each movement of the clasp becomes painful; and when that happens the denture ceases to function as a masticating unit—it may be worn, but it is not used.

Another phase of high importance is the resorption that may occur. When resorption occurs occlusion is lost. In the construction of gold cases this should be remembered, and when possible, provision made to allow for rebasing. A restoration can be made that will help to promote a normal condition of bone and tissue life, and by the same token, a denture can cause the excessive resorption of tissue and bone.

This question is one that few men agree upon; what one dentist says wil cause resorption, another will say maintains normal conditions. Some men will prove that pressure causes resorption, others will prove that pressure promotes a normal condition. They are both right, and the apparent difference in their opinions is explained by the different types of pressure that they have in mind. For instance, a continuous pressure that stops the flow of either venous or arterial blood will cause resorption, but a pressure that has a pounding effect, one that is not continuous, will have a stimulating effect and promote normal blood flow. When the blood flow can be maintained in a normal degree, there will be very little resorption. Resorption will always be in harmony with the diminishing of the blood flow.

It follows then when the teeth are extracted and the jaws are not called upon to do much work, the diminished blood flow results in the consequent resorption. But if a comfortable denture is then placed in the mouth, one that the patient will chew on, the pounding effect will stimulate the blood flow and resorption will be checked. In fact if it were possible to use the denture enough to maintain a normal flow of blood, there would be no resorption at all.

A correct understanding of the two kinds of pressure that a denture can exert is especially needed by dentists who use what is known as "the biting stress relation." A number of years ago Prof. Peeso, of Philadelphia, first introduced a system of bridge-work that placed sufficient pressure upon the jaw to promote blood flow, and thus minimize resorption. His system was based upon the use of retaining agents that could move toward the jaw during the displacement of mucosa. In that manner the masticating pressure was carried directly to the jaw underlying the bridge. While the jaws were at rest the bridge lightly rested upon tissue, but when mastication commenced the pressure was placed directly on the jaw, because the retaining agents allowed of a movement in harmony with the displacement of tissue. To determine the correct relationship of the various parts, Dr. Peeso took what was termed a "biting stress relation." That is, the relation of the saddle to the retaining agents, was obtained under biting stress with the mucosa displaced. But when the bridge was placed in the mouth the saddle did not displace tissue except during mastication.

Since the advent of the cast clasp, a great many men use the Peeso biting stress relation to overcome the displacement of mucosa. But they do not have movable retaining agents such as Dr. Peeso used, but a fixed cast clasp, and of course an altogether different effect. When the biting stress relation is used with fixed cast clasps, the mucosa is continually displaced because there is no opportunity for a release, the clasps being fixed. This continual pressure stops the flow of venous blood, which reacting on the arterial flow soon causes resorption. And until the resorption takes place the denture is uncomfortable because the abutment tooth is continually called upon to supply the force which keeps the mucosa continually displaced. In other words, in order to remove stress during mastication, we have a continual stress all the time the jaws are at rest.

This same effect is seen when compound impressions are used in connection with cast-clasp dentures, or partials with any type of fixed retaining agents. It is absolutely impossible to take a compound impression without displacing tissue. In scientific impression-taking for full dentures modelling compound is used because tissue can be displaced with it. When compound is confined in a tray, and sufficient pressure used to take the impression, some of the tissue is always displaced. But when a biting block is used in taking the impression, the maximum displacement of mucosa takes place.

When the impression is for a full denture the biting-block displacement effect is only noticed during mastication. At all other times the denture is just resting against the tissue to the extent that atmospheric pressure holds it there. But when a biting stress compound impression is used for a partial case with fixed attachments we have an altogether different effect. When the biting block impression is taken the mucosa is displaced, but the abutment teeth remain stationary, and of course the position of the retaining agent as well. So then the finished denture when it is placed in the mouth will continually displace as much mucosa as was displaced when the impression was taken. As the abutment teeth do not move, and as the fixed attachments do not move, relation of the attachments to the tissue will be just the same when the denture is in position as when the impression was taken. So if mucosa is displaced during the taking of the impression it will surely be continually displaced when the denture is in position. This of course is another cause of sore abutments, uncomfortable restorations and resorption.

To sum up then, the ideal partial denture will first of all have occlusion. It will be held in place by retaining agents that will properly retain it, without placing undue stress on the abutment teeth. The retaining agents will allow the denture to move with the displacement of mucosa so as to have a pounding effect on the tissue, and promote blood flow. The retaining agents will not cause binding or uncomfortable tight feeling. And last of all, the impression upon which the denture is built, must be of plaster, so that the mucosa will not be displaced. With these conditions carried out, partial dentures will restore occlusion, will be tight and will be comfortable. Furthermore, such a denture will have a good chance of remaining tight through the minimizing of resorption, and by the same means will remain in occlusion and be comfortable.

Pyorrhea specialists are frank to admit that a great deal of pyorrhea is caused by the lack of occlusion, where dentures are worn but not used, resulting in over-functioning of other teeth. In some instances dentures badly needed are not worn at all, because the dentist had no hope of making a satisfactory restoration. It is to be hoped that with a broader knowledge of the subject, partial denture work will soon be on a par with other branches of prosthetic dentistry.—*Dental Summary*, for August, 1921.

